# Correction to “Association Between CD20+ T Lymphocytes and Neuropsychological Findings in Multiple Sclerosis”

**DOI:** 10.1111/ene.70258

**Published:** 2025-08-21

Esposito A, Falco F, Scalia G, et al. Association between CD20+ T lymphocytes and neuropsychological findings in multiple sclerosis. *Eur J Neurol*. 2025; 32:e16536. https://doi.org/10.1111/ene.16536

In the Results section of the abstract, the number of patients reported was incorrect in the sentence “Seventy patients (18.9%) showed CD20+ T lymphocytes in peripheral blood with a mean level of 0.38 ± 1.2%.”

It should have been: “Seventeen patients (18.9%) showed CD20+ T lymphocytes in peripheral blood with a mean level of 0.38 ± 1.2%.”

We apologize for this error.

